# Prognostic value of cancer-related fatigue at the end of radiotherapy for overall survival ≥ 10 years in women with breast cancer

**DOI:** 10.1186/s13058-025-02036-3

**Published:** 2025-05-12

**Authors:** Philipp Heumann, Axel Benner, Sabine Behrens, Jenny Chang-Claude, Petra Seibold

**Affiliations:** 1 Division of Cancer Epidemiology, German Cancer Research Center (DKFZ), Im Neuenheimer Feld 280, 69120 Heidelberg, Germany; 2 Medical Faculty Heidelberg, Heidelberg University, Heidelberg, Germany; 3 Division of Biostatistics, German Cancer Research Center (DKFZ), Heidelberg, Germany; 4 University Cancer Center Hamburg, University Medical Center Hamburg-Eppendorf, Hamburg, Germany

**Keywords:** Breast neoplasms, Fatigue, Survival, Radiotherapy, Patient reported outcome measures

## Abstract

**Background:**

Cancer-related fatigue (CRF) is a common symptom in breast cancer patients and survivors, which can substantially impair quality of life. Previous studies suggested that CRF may be associated with poorer survival outcomes, but had limited follow-up duration or insufficient adjustment for established prognostic factors. The aim of this analysis was to assess the prognostic value of CRF at the end of radiotherapy for overall survival in a cohort of women with breast cancer with a median follow-up time of 19 years.

**Methods:**

Data from the prospective *ISE* study, which enrolled women with non-metastatic breast cancer between 1998 and 2001, were analysed. Patients did not receive chemotherapy. A vital status follow-up was conducted in 2019. CRF was collected at the end of radiotherapy using the EORTC QLQ-C30 and classified using the threshold of clinical importance. Cox regression models adjusted for CRF, age, body mass index (BMI), tumour size, nodal involvement, grading and receptor status were calculated.

**Results:**

Of 437 patients with fatigue assessments, 164 (38%) reported CRF. During 10 years of follow-up, 25 patients without and 27 patients with CRF died. Tumour size, nodal involvement and age were statistically significantly associated with 10-year overall survival. For CRF, a statistically significant effect was observed for ≥ 5 years of follow-up (HR: 2.44), but not within the first 5 years of follow-up (HR: 1.26).

**Conclusions:**

CRF assessments at the end of radiotherapy showed prognostic value for long-term survival beyond established factors and could potentially be used to identify patients that require monitoring in risk-adapted aftercare programmes in order to improve survival.

**Supplementary Information:**

The online version contains supplementary material available at 10.1186/s13058-025-02036-3.

## Introduction

Cancer-related fatigue (CRF) is a common symptom experienced by patients and survivors at different stages of the disease, from diagnosis and treatment to years after completion of treatment [1]. CRF is defined as ’a distressing, persistent, subjective sense of physical, emotional, and/or cognitive tiredness or exhaustion related to cancer or cancer treatment that is not proportional to recent activity and interferes with usual functioning’ [2]. It has been estimated that 22–53% of breast cancer patients experience moderate to severe CRF at the end of treatment [3–5]. A meta-analysis of more than 12,000 breast cancer survivors found prevalences of severe CRF ranging from 7 to 52%, with proportions varying considerably depending on the treatment received and the instrument used to measure CRF [6]. Although the aetiology and pathogenesis of CRF remains partly unknown, several pathways have been hypothesised, such as dysregulation of cytokines [7]. Despite a growing body of evidence pointing to the role of proinflammatory cytokines, including C-reactive protein and tumour necrosis factor alpha, it is unclear whether they cause or exacerbate CRF [8].

Although guidelines recommend CRF screening during treatment and aftercare, there remains potential for further improvements in clinical implementation [9]. In a recent study, 41% of cancer survivors reported that they were never asked about their exhaustion by their treating physicians [10]. Thus, part of the patient’s symptom burden may not be detected, impeding comprehensive symptom management.

CRF as a subjective experience with multiple clinical manifestations is commonly assessed using patient self-reports [1, 11]. Patient-reported CRF assessments may provide additional benefits beyond capturing the symptom at the time of assessment. Previous studies suggest that CRF may be associated with poorer overall survival in breast cancer patients, although results are limited by short follow-up times or insufficient adjustment for established prognostic factors [12–15].

Therefore, the aim of this study was to investigate the prognostic value of clinically important CRF at the end of radiotherapy for overall survival in a cohort of breast cancer patients, accounting for established prognostic factors. In addition, Global Health Status/Quality of Life (GHS/QoL) was also examined as a potential prognostic marker in order to determine whether an association between CRF and overall survival reflects more general differences in a broader domain of patient well-being.

## Methods

Data were derived from the prospective cohort study *ISE* on acute and long-term toxicities of breast radiotherapy [16, 17]. 478 women with histologically confirmed breast cancer or in situ carcinoma were recruited after breast-conserving surgery between 1998 and 2001 in the radiation oncology departments of the Women’s Hospital in Heidelberg, the St. Vincentius Hospital and the City Hospital in Karlsruhe and the University Medical Centre Mannheim. Patients received whole-breast radiotherapy with 50 Gy (2 Gy/fraction) or 50.4 Gy (1.8 Gy/fraction) followed by a percutaneous (5–20 Gy) or brachytherapy (20–25 Gy) boost, alternatively 56 Gy (2 Gy/fraction) without boost. Patients treated with chemotherapy were not eligible. Women with second primary malignancy, bilateral breast tumours and/or metastases before radiotherapy initiation were excluded. Written informed consent was obtained from all patients. The ethics committee of the University of Heidelberg approved the study. The study was performed in accordance with the Declaration of Helsinki.

Demographic characteristics, medical history, family history of cancer, comorbidities and lifestyle factors were recorded by patients using a questionnaire prior to radiotherapy. Tumour and treatment data were extracted from medical records. A vital status follow-up was conducted in mid-2019 via record linkage with local residents’ registration offices. Death certificates were obtained from local health authorities. Patients were prospectively asked to rate their health-related quality of life (HRQoL) using the EORTC QLQ-C30 questionnaire at the start of adjuvant radiotherapy, after receiving 36–42 Gy and 44–50 Gy, at radiotherapy completion, and at their first aftercare visit up to 8 weeks later [18]. The EORTC QLQ-C30 consists of multi-item symptom and functional scales, a global health scale and individual symptoms commonly reported by cancer patients. Standardised scores ranging from 0 to 100 were calculated for the fatigue scale used to define CRF (items 10, 12, 18) and for the GHS/QoL scale (items 29, 30), with higher values indicating higher symptom levels or higher QoL [19]. Prevalent CRF was defined based on the threshold for clinically important levels as fatigue scores ≥ 39, although this definition was not equivalent to a clinical diagnosis [20]. As there is no commonly used threshold for the GHS/QoL scale, scores were categorised into tertiles, with the lower tertile defined as clinically important impairment.

For this analysis, patients’ scores at the first aftercare visit after radiotherapy completion were used to define CRF and GHS, and henceforth termed ‘CRF or GHS at the end of radiotherapy‘. Last observations were carried forward by substituting missing assessments at the aftercare visit by those at radiotherapy completion (18%). Overall survival, which constitutes the time from HRQoL questionnaire assessment at the end of radiotherapy to death from any cause, was defined as the endpoint. As primary analysis, the prognostic value of CRF for overall survival with a follow-up of 10 years was assessed, thus events after 10 years of follow-up were censored at 10 years. Kaplan-Meier curves stratified by CRF were generated to estimate distributions of overall survival. Median follow-up time was calculated using the reverse Kaplan-Meier method [21]. Multivariable Cox proportional hazards models adjusted for the candidate prognostic factors age, body mass index (BMI), tumour size, lymph node involvement, grading and hormone receptor positivity were conducted to assess the prognostic value of CRF on overall survival. As secondary analysis, the association of CRF with overall survival after 15 years follow-up was investigated. Additionally, the association between the GHS/QoL score with 10- and 15-year overall survival, respectively, was examined, comparing the lowest vs. the intermediate and highest tertiles using multivariable Cox proportional hazard models adjusted for the aforementioned covariates. As the evaluation of scaled Schoenfeld residuals indicated non-proportional hazards for CRF, the Cox models were extended with a step function at 5 years of follow-up (Appendix A).

Tumour size (In situ/TX/T0/T1 vs. T2–T4), lymph node involvement (NX/N0 vs. N1/N2), and hormone receptor positivity were included dichotomised in the models. Hormone receptor positivity was determined by oestrogen and/or progesterone receptor status. If oestrogen and/or progesterone status were indeterminate and/or missing, positivity was assigned based on receipt of hormone therapy. Grading was included as an ordinal variable. Age and BMI were modelled as continuous covariates, with missing values substituted by data from the first *ISE* follow-up from 2003 to 2005 [22]. Statistical analyses were performed using R 4.2.2 and the package survival [23, 24].

## Results

Of 478 breast cancer patients in the *ISE* cohort, 454 provided a fatigue assessment at the end of radiotherapy. 9 patients were excluded due to a second primary malignancy, 6 due to bilateral disease and 2 due to metastases at radiotherapy initiation. The median age of the 437 patients included was 60 years, with the youngest woman being 26 years and the oldest 87 years old (Table 1).

**Table 1:** Selected characteristics of 437 breast cancer patients of the *ISE* cohort, additionally stratified by clinically important levels of cancer-related fatigue (CRF; ≥39 scores, based on the EORTC QLQ-C30) at the end of radiotherapy

Characteristic	No CRF, *N* = 273^1^	CRF, *N* = 164^1^	Overall, *N* = 437^1^
**Deceased From Any Cause**			
10-year Follow-Up	25 (9.2%)	27 (16.5%)	52 (11.9%)
15-year Follow-Up	45 (16.5%)	41 (25%)	86 (19.7%)
**Age (years)**			
Mean (SD)	60 (9)	61 (9)	61 (9)
Range	26–87	38–85	26–87
**Body Mass Index**			
< 25 kg/m²	132 (49%)	65 (40%)	197 (45%)
25–30 kg/m²	113 (42%)	70 (43%)	183 (42%)
> 30 kg/m²	27 (9.9%)	28 (17%)	55 (13%)
Missing	1	1	2
**Tumour Size**			
T0	1 (0.4%)	0	1 (0.2%)
T1	181 (66%)	112 (68%)	293 (67%)
T2	64 (23%)	41 (25%)	105 (24%)
T4	1 (0.4%)	0	1 (0.2%)
TX	0	1 (0.6%)	1 (0.2%)
In Situ	26 (9.5%)	10 (6.1%)	36 (8.2%)
**Nodal Status**			
N0	208 (76%)	125 (76%)	333 (76%)
N1	38 (14%)	27 (16%)	65 (15%)
N2	1 (0.4%)	1 (0.6%)	2 (0.5%)
NX	26 (9.5%)	11 (6.7%)	37 (8.5%)
**Histological Type**			
In Situ	28 (10%)	9 (18%)	37 (8.5%)
Invasive Ductal	152 (56%)	100 (61%)	252 (58%)
Invasive Lobular	57 (21%)	32 (20%)	89 (20%)
Other	36 (13%)	23 (14%)	59 (14%)
**Histological Grading**			
Grade 1	50 (19%)	29 (18%)	79 (19%)
Grade 2	169 (64%)	101 (63%)	270 (64%)
Grade 3	46 (17%)	30 (19%)	76 (18%)
Missing	8	4	12
Hormone Receptor Status (ER/PR)*			
Negative	32 (12%)	10 (6.1%)	42 (9.7%)
Positive	240 (88%)	153 (94%)	393 (90%)
Missing	1	1	2
**Total Radiotherapy Dose**			
50–60.4 Gy	150 (55%)	104 (63%)	254 (58%)
> 60.4 Gy	123 (45%)	60 (37%)	183 (42%)

^1^n (%); *Positive receptor status indicates oestrogen receptor and/or progesterone receptor positivity. If oestrogen receptor and/or progesterone receptor status were indeterminate and/or missing, positivity was determined based on receipt of hormone therapy

**Fig. 1:**
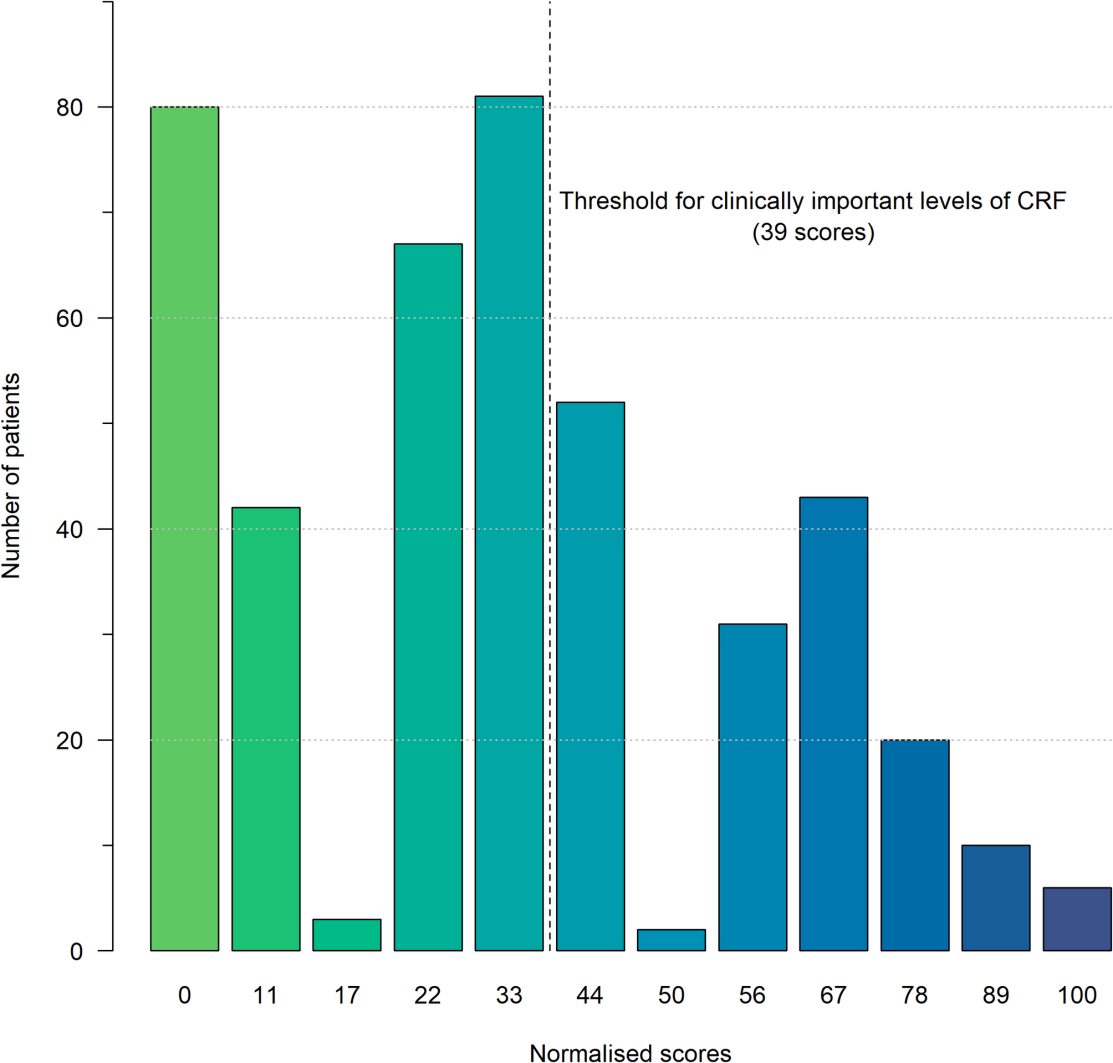
Absolute frequency of cancer-related fatigue (CRF) scores at the end of radiotherapy of 437 included breast cancer patients of the *ISE* cohort along with the threshold for clinically important scores

Most patients had early stage disease. 293 women (67%) had a tumour < 2 cm and 333 (76%) did not have regional lymph node metastases. The median duration between radiotherapy completion and HRQoL questionnaire assessment was 26 days (IQR: 36). 164 patients (38%) reported prevalent clinically relevant CRF (scores ≥ 39) at the end of radiotherapy. The median CRF score was 33 (IQR: 44), which fell just below the threshold for clinically important levels (Fig. 1).

The median GHS score was 67 (IQR: 33; Appendix B). Fatigued women were more frequently obese (17% vs. 9%) and had hormone receptor status positive tumours (94% vs. 88%), whereas no substantial differences were observed for tumour size, nodal involvement and grading.

**Fig. 2:**
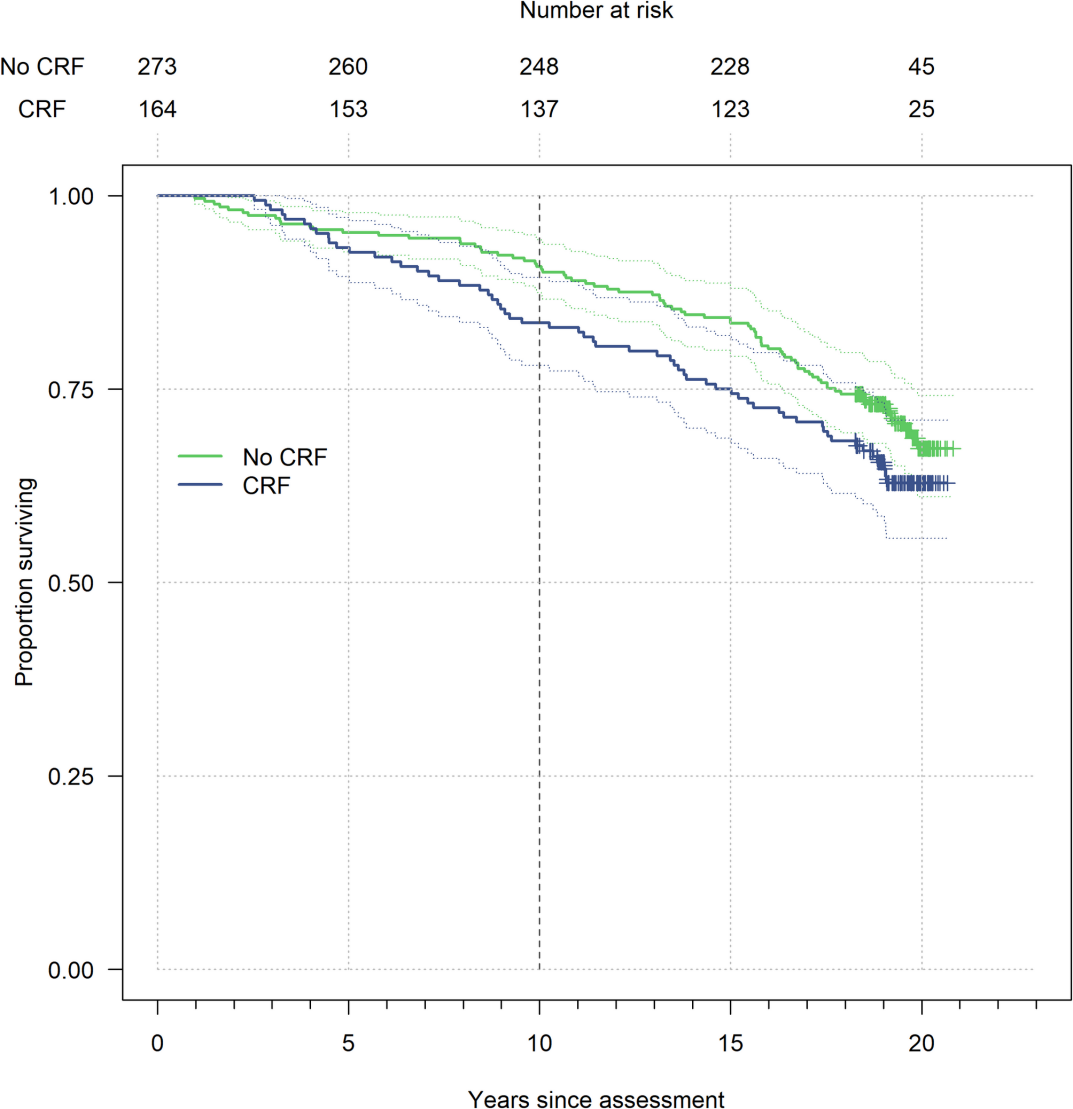
Kaplan-Meier curves for 437 included breast cancer patients of the *ISE* cohort stratified by clinically important levels of cancer-related fatigue (CRF; ≥39 scores, based on the EORTC QLQ-C30) at the end of radiotherapy

During the overall follow-up period with a median of 19 years, 139 patients died, corresponding to 36% of those with and 29% without CRF. Of the 52 patients who died within the first 10 years of follow-up, 27 (48%) reported clinically important CRF levels at the end of radiotherapy. Kaplan-Meier curves showed minor differences for the first years of follow-up with a slightly higher survival rate for patients who had previously reported fatigue (Fig. 2). The survival curves diverged at about 5 years of follow-up and showed with longer follow-up a consistently higher survival rate for non-fatigued patients. This trend persisted over the remaining follow-up period up to more than 20 years post-radiotherapy.

In the multivariable model, 421 patients with complete data were included (Table 2). Tumour size (HR: 3.02, *P* < 0.005), nodal involvement (HR: 2.56, *P* < 0.005) and age (HR: 1.05, *P* < 0.005) were statistically significant prognostic factors for overall survival. For CRF, a statistically significant association with overall survival was observed beyond 5 years of follow-up, indicating that fatigued patients had a 2.44-fold higher hazard (95% CI: 1.12 to 5.32) of dying from any cause compared to patients who did not report CRF at the end of radiotherapy. The association was weaker for the first 5 years of follow-up with an HR of 1.26 (95% CI: 0.56 to 2.84) and not statistically significant. All other covariables did not show a significant association, including BMI, grading and hormone receptor positivity.

**Table 2:** Hazard ratios and 95% confidence intervals of candidate prognostic factors of the multivariable Cox proportional hazards models on overall survival of 421 breast cancer patients of the *ISE* cohort

	10-year Follow-Up		15-year Follow-Up	
	Hazard Ratio (95% CI)	*P*	Hazard Ratio (95% CI)	*P*
Cancer-related Fatigue*				
< 5 Years of Follow-Up	1.26 (0.56 to 2.84)	0.57	1.29 (0.57 to 2.90)	0.54
≥ 5 Years of Follow-Up	2.44 (1.12 to 5.32)	0.02	1.64 (0.97 to 2.77)	0.07
**Tumour Size** (T2–T4 vs. in Situ/T0/T1/TX)	3.02 (1.69 to 5.38)	< 0.005	1.99 (1.24 to 3.20)	< 0.005
**Nodal Involvement** (N1/N2 vs. N0/NX)	2.56 (1.41 to 4.65)	< 0.005	1.89 (1.14 to 3.14)	0.01
**Grading**				
Grade 1	Reference			
Grade 2	0.69 (0.32 to 1.49)	0.34	0.77 (0.42 to 1.40)	0.40
Grade 3	0.77 (0.31 to 1.94)	0.58	0.79 (0.38 to 1.65)	0.54
**Hormone Receptor Positivity (ER/PR)**	0.46 (0.19 to 1.11)	0.08	0.47 (0.24 to 0.95)	0.04
**Age**	1.05 (1.02 to 1.09)	< 0.005	1.09 (1.06 to 1.11)	< 0.005
**Body Mass Index** (kg/m^2^)	1.05 (0.99 to 1.12)	0.13	1.02 (0.96 to 1.07)	0.54

*Cancer-related fatigue was defined based on standardised scores of the EORTC QLQ-C30 fatigue scale (items 10, 12, 18) and the proposed threshold for a clinically important CRF levels as scores ≥ 39.

### Secondary analyses

The secondary analysis of overall survival with 15 years of follow-up included 86 patients who died during this period, corresponding to 25% of fatigued and 16% of non-fatigued women. Tumour size (HR: 1.99, *P* < 0.005), nodal involvement (HR: 1.89, *P* = 0.01) and age (HR: 1.09, *P* < 0.005) remained associated with poorer overall survival (Table 2) whereas hormone receptor positivity was associated with improved overall survival (HR: 0.47, *P* = 0.04). CRF was associated with reduced overall survival up to 5 years (HR: 1.29, 95% CI: 0.57 to 2.90) as well as beyond (HR: 1.64, 95% CI: 0.97 to 2.77), albeit not statistically significant. Estimates of the other candidate prognostic factors did not change substantially. GHS was not statistically significantly associated with hazard (Table 3).

**Table 3:** Hazard ratios and 95% confidence intervals of candidate prognostic factors of the multivariable Cox proportional hazards models on overall survival of 417 breast cancer patients of the *ISE* cohort

	10-year Follow-Up		15-year Follow-Up	
	Hazard Ratio (95% CI)	*P*	Hazard Ratio (95% CI)	*P*
Global Health Status/Quality of Life* (lowest tertile vs. intermediate and highest tertiles)	1.60 (0.91 to 2.82)	0.10	1.52 (0.97 to 2.37)	0.07
**Tumour Size**	2.94 (1.65 to 5.25)	< 0.005	1.98 (1.24 to 3.18)	< 0.005
**Nodal Involvement**	2.64 (1.45 to 4.83)	< 0.005	1.93 (1.16 to 3.21)	0.01
**Grading**				
Grade 1	Reference			
Grade 2	0.72 (0.33 to 1.57)	0.41	0.80 (0.44 to 1.45)	0.46
Grade 3	0.89 (0.35 to 2.24)	0.81	0.86 (0.41 to 1.80)	0.69
**Hormone Receptor Positivity (ER/PR)**	0.52 (0.21 to 1.27)	0.15	0.52 (0.26 to 1.05)	0.07
**Age**	1.04 (1.01 to 1.08)	0.008	1.08 (1.05 to 1.11)	< 0.005
**Body Mass Index** (kg/m^2^)	1.06 (0.99 to 1.13)	0.07	1.02 (0.97 to 1.08)	0.39

*Global Health Status/Quality of Life was defined based on standardised scores of the EORTC QLQ-C30 Global Health Status/Quality of Life scale (items 29, 30), with the lowest tertile defined as clinically important impairment and compared with the intermediate and highest tertile

### Sensitivity analysis

The time point of data collection at the end of radiotherapy varied in the sample. CRF was assessed by 21 women up to 18 days before and by 35 women between 8 and 16 weeks after radiotherapy completion. In a sensitivity analysis, the patient population was restricted to 381 women with CRF assessments between radiotherapy completion and up to 8 weeks afterwards. Tumour size (HR: 3.35, *P* < 0.005), nodal involvement (HR: 2.53, *P* < 0.005) and age (HR: 1.05, *P* = 0.007) remained associated with overall survival given 10 years of follow-up (Appendix C). Consistent with the primary analysis, CRF showed a statistically significant association with overall survival after 5 years of follow-up (HR: 2.52, 95% CI: 1.13 to 5.62). After stratification by median age, survival curves for both women younger and older 60 years showed with longer follow-up a higher survival rate for those without CRF (Appendix D1). In the multivariable Cox model, a statistically significant association was observed in women younger than 60 years between CRF beyond 5 years of follow-up and 10-year overall survival (Appendix D2).

## Discussion

This study aimed to investigate the prognostic value of CRF at the end of radiotherapy for overall survival beyond established prognostic factors in breast cancer patients. CRF was found to be a statistically significant predictor of overall survival in breast cancer patients treated with radiotherapy after ≥ 5 years of follow-up, in addition to tumour size, lymph node involvement and age, considering 10-year follow-up. Stratified analyses showed that this association was strongest in women younger than 60 years at radiotherapy.

These results are consistent with studies that provided initial evidence of lower survival in patients experiencing CRF. Comparisons, however, are limited by differences in disease and treatment characteristics, as well as the operationalisation of CRF and corresponding thresholds, and statistical modelling approaches. Previous univariate analyses showed a lower survival rate for fatigued breast cancer patients with advanced disease, which was, however, not confirmed in multivariable analyses or only for a limited median follow-up of 5 years [13, 14, 25]. A statistically significant association between CRF at the end of radiotherapy and recurrence free survival has also been reported [12].

To the best of the authors’ knowledge, our study is the first to demonstrate the prognostic value of CRF at the end of local treatment for overall survival in non-metastatic breast cancer after accounting for established prognostic factors. CRF in patients was defined based on the proposed threshold for clinically important levels of fatigue, as opposed to sample-based cut-offs.

The findings show an additional benefit of CRF assessments beyond symptom detection, which could facilitate early identification of patients with poorer survival outcomes up to 10 years after treatment. Risk-adapted aftercare programmes could offer closer monitoring of affected patients and assist in identifying indications of decreased survival outcomes with particular focus on patients younger than 60 years. Before recommendations for action are proposed, external validation of these findings in larger cohorts of breast cancer patients are necessary. Studies should also include patients who received more modern treatments and have used other CRF measurement tools.

According to the objectives of this study, CRF was investigated as an independent prognostic factor, irrespective of underlying causal pathways. Yet, a potential causal mechanism for the observed relationship between CRF and overall survival could be hypothesised. A recent study found that 31% of fatigued breast cancer patients at the end of treatment experienced CRF more than 5 years later [26]. Assuming that a substantial proportion of fatigued patients at the end of radiotherapy experienced persistent CRF, reduced adherence to aftercare visits or uptake of other health services due to severe fatigue might contribute to the observed survival differences. Indeed, the observed differences in survival rates became apparent only after several years. On the other hand, there was no statistically significant CRF effect in the 15-year follow-up analysis. GHS/QoL scores, as a broader indicator of well-being, did not emerge as a prognostic factor of overall survival, although *P* values were small. 61% of patients in the *ISE* cohort who reported being fatigued 10 years later indicated prevalent CRF at the end of radiotherapy (data not shown).

The generalisability of the results is limited as only women with early breast cancer who did not receive chemotherapy were included. As 60% of early breast cancer patients do not receive chemotherapy as part of their initial treatment, a substantial proportion of the patient population is nevertheless represented, although generalisability with regard to advanced disease may be limited [27]. HER2 status of the patients was not available, as recruitment took place before routine testing was implemented. Tumour size and lymph node involvement were not available as continuous variables, whereby both covariates were analysed dichotomised due to small group sizes (1 patient with T4, 2 patients with N2). Furthermore, local therapies were administered according to standards at the time of patient inclusion around 20 years ago. 18% of the analysed CRF assessments were not collected at the first aftercare visit after radiotherapy, but at the earlier time point of radiotherapy completion. Although the agreement between the two time points was 75%, this may have led to some misclassification of fatigued and non-fatigued patients.

Among the strengths of this analysis are the extensive prospective data collection that allowed the long-term prognostic value of CRF to be investigated and the standardised assessment of CRF using the validated and widely used EORTC QLQ-C30. Moreover, patients were categorised according to the proposed threshold for clinically important levels of fatigue. Further prognostic factors included in the statistical modelling were selected under consideration of their availability in clinical practice and constrained by the data collected in *ISE*. This resulted in a parsimonious statistical model accounting for the most commonly used prognostic factors in breast cancer [28].

In conclusion, the results suggest that patient-reported CRF at the end of radiotherapy can provide prognostic benefit for overall survival in breast cancer patients beyond established prognostic factors. Further implementation of recommended routine fatigue screenings could help identify patients that require monitoring in risk-adapted aftercare programmes in order to improve survival outcomes.

## Electronic supplementary material

Below is the link to the electronic supplementary material.

Supplementary Material 1

## Data Availability

The datasets analysed in this study are available on reasonable request from the corresponding author.

## Abbreviations

95% CI: 95% confidence interval
BMI: Body mass index
CRF: Cancer-related fatigue
GHS: Global health status
Gy: Gray
HR: Hazard ratio
HRQoL: Health-related quality of life
QoL: Quality of life
