# Supplementary material for: Prognostic value of cancer-related fatigue at the end of radiotherapy for overall survival ≥ 10 years in women with breast cancer
Source: Breast Cancer Res. 2025 May 12;27:76. doi: 10.1186/s13058-025-02036-3 (PMC12070715; doi:10.1186/s13058-025-02036-3)
Supplement: Supplementary file 1 — Supplementary Material 1 [file 13058_2025_2036_MOESM1_ESM.docx]

## Appendix

###
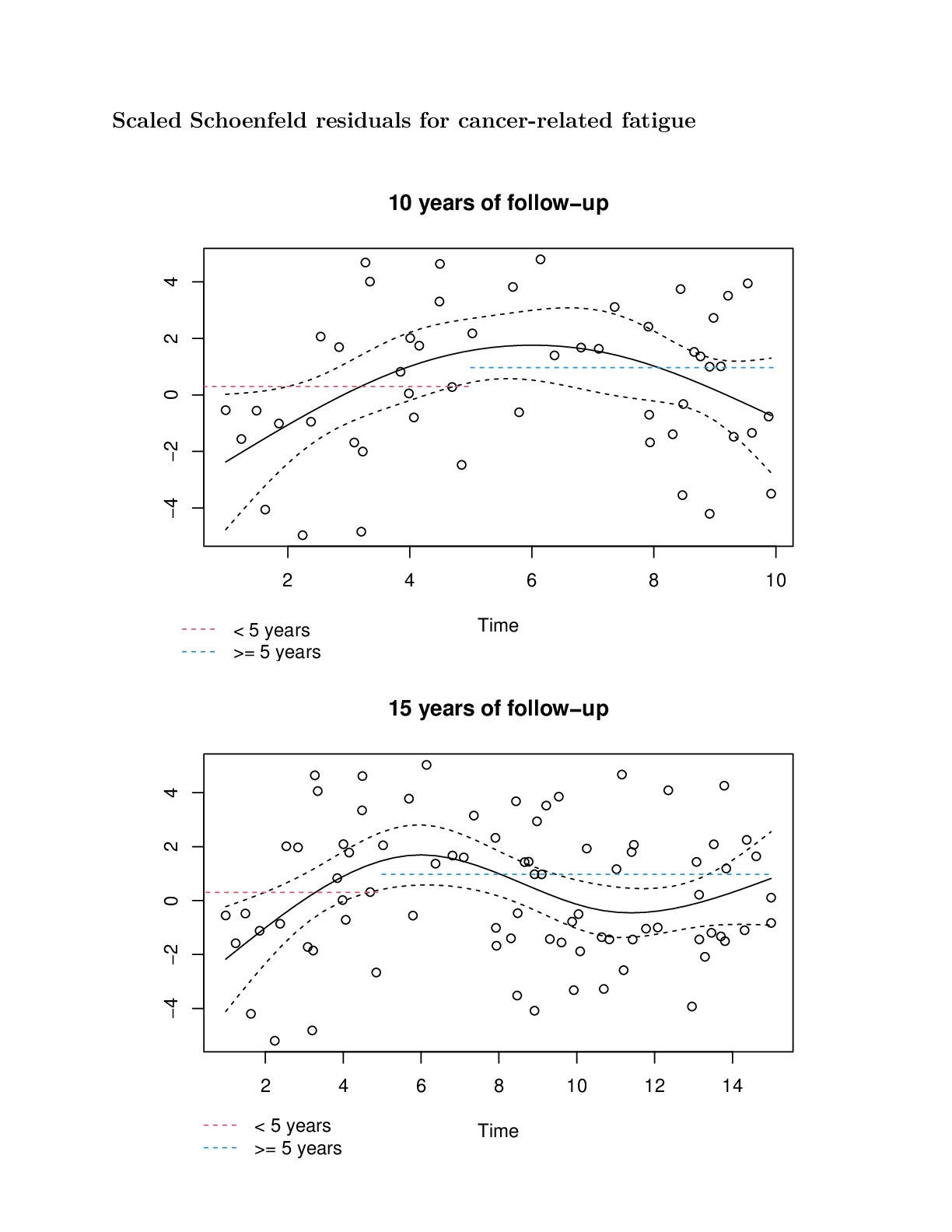
A

###
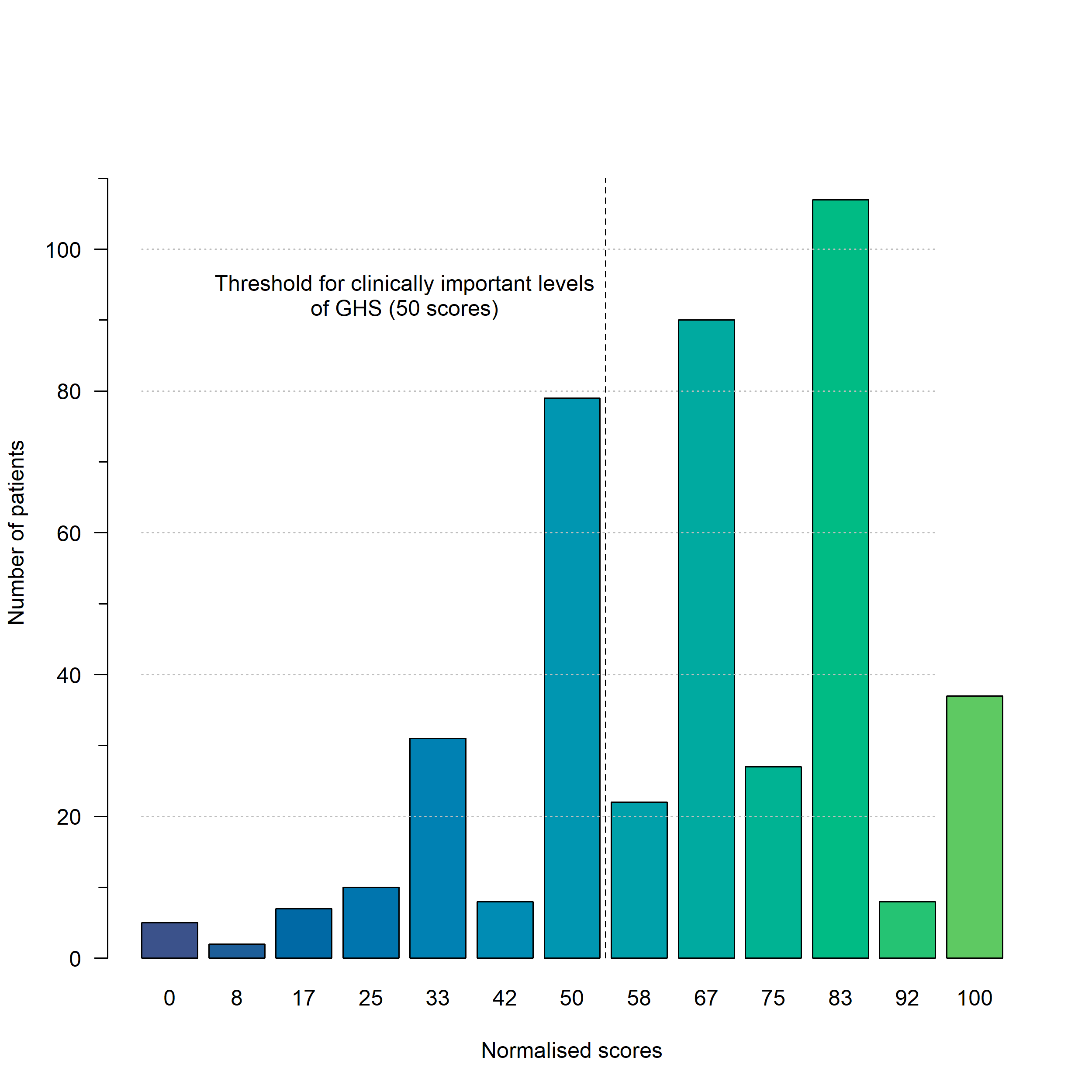
B

Absolute frequency of Global Health Status/Quality of Life scores at the end of radiotherapy of 433 included breast cancer patients of the *ISE* cohort along with the threshold for clinically important impairment, defined as the lowest tertile.

### C

Hazard ratios and 95% confidence intervals of candidate prognostic factors of the multivariable Cox proportional hazards models on overall survival of 369 breast cancer patients of the *ISE* cohort with cancer-related fatigue assessments between radiotherapy completion up to 8 weeks afterwards.

|  | **10-year  Follow-Up** |  |
| --- | --- | --- |
|  | Hazard Ratio (95% CI) | *P* |
| **Cancer-related Fatigue*** |  | |
| <5 Years of Follow-Up | 0.97 (0.41 to 2.28) | 0.94 |
| ≥5 Years of Follow-Up | 2.52 (1.13 to 5.62) | 0.02 |
| **Tumour Size** (T2–T4 vs. in Situ/T0/T1/TX) | 3.35 (1.85 to 6.07) | <0.005 |
| **Nodal Involvement** (N1/N2 vs. N0/NX) | 2.53 (1.36 to 4.71) | <0.005 |
| **Grading** |  | |
| Grade 1 | Reference | |
| Grade 2 | 0.60 (0.27 to 1.32) | 0.20 |
| Grade 3 | 0.70 (0.28 to 1.76) | 0.45 |
| **Hormone Receptor Positivity (ER/PR)** | 0.46 (0.19 to 1.11) | 0.08 |
| **Age** | 1.05 (1.01 to 1.08) | 0.007 |
| **Body Mass Index** (kg/m^2^) | 1.07 (1.00 to 1.14) | 0.06 |
| *Cancer-related fatigue was defined based on standardised scores of the EORTC QLQ-C30 fatigue scale (items 10, 12, 18) and the proposed threshold for a clinically important CRF levels as scores ≥39. | | |

###
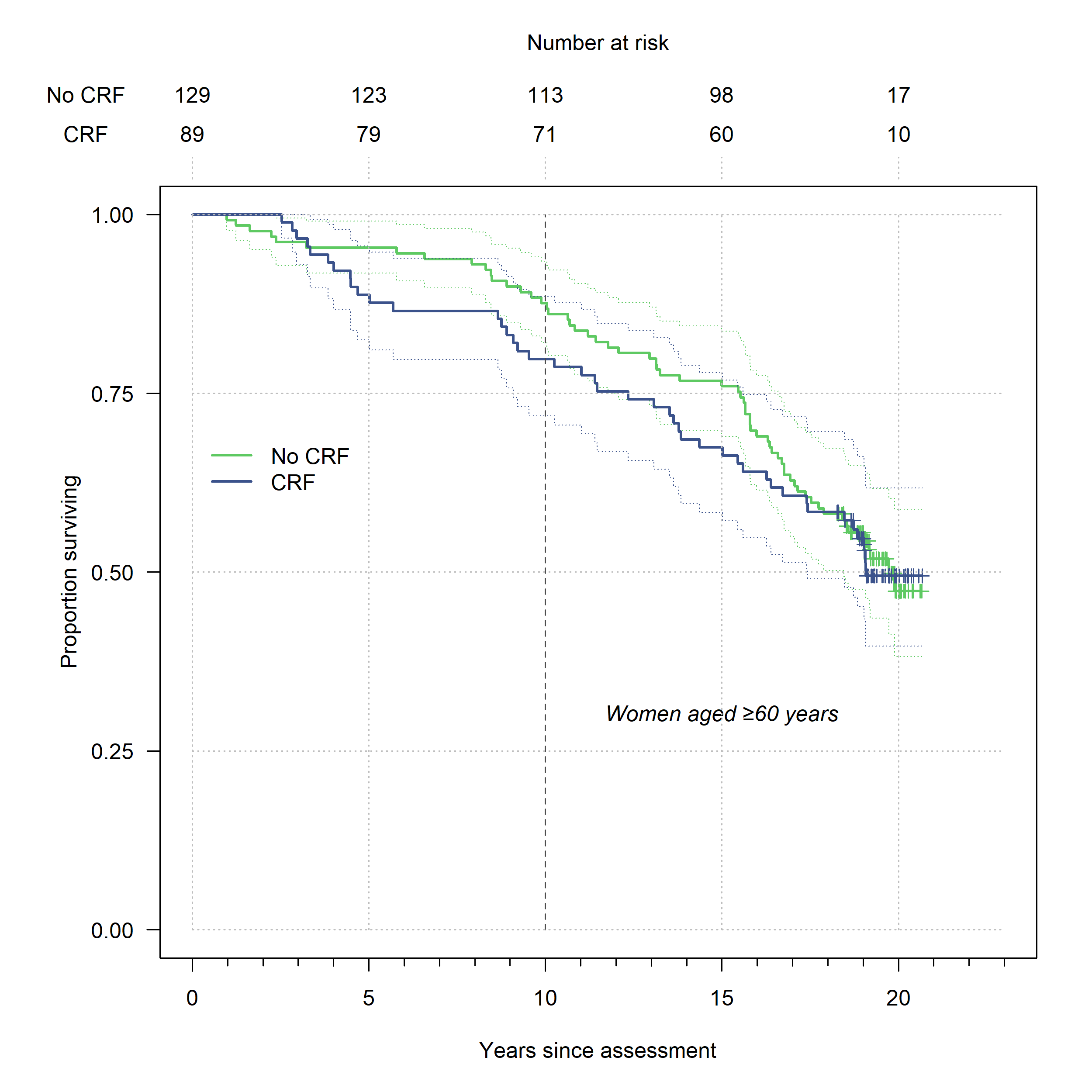

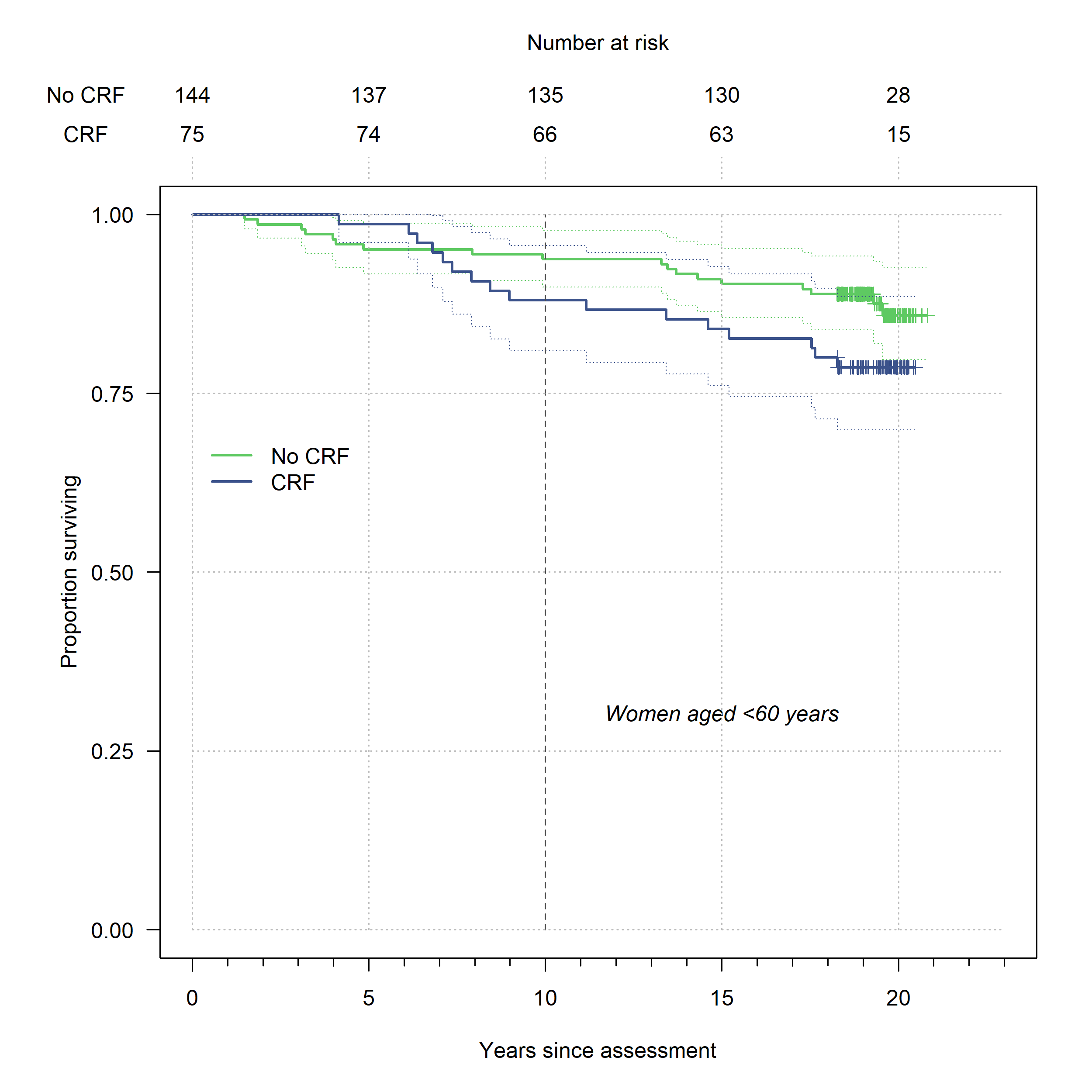
D1

Kaplan-Meier curves for breast cancer patients of the ISE cohort stratified by median age (219 women aged <60 years vs. 218 women aged ≥60 years) and clinically important levels of cancer-related fatigue (CRF; ≥39 scores, based on the EORTC QLQ-C30) at the end of radiotherapy.

### D2

Hazard ratios and 95% confidence intervals of candidate prognostic factors of the multivariable Cox proportional hazards models on overall survival of 421 breast cancer patients of the *ISE* cohort stratified by age.

|  | **10-year  Follow-Up** | | | |
| --- | --- | --- | --- | --- |
|  | *<60 years (211 women)* | | *≥60 years (210 women)* | |
|  | Hazard Ratio (95% CI) | *P* | Hazard Ratio (95% CI) | *P* |
| **Cancer-related Fatigue*** |  | |  |  |
| <5 Years of Follow-Up | 0.27 (0.03 to 2.25) | 0.23 | 2.11 (0.76 to 5.91) | 0.15 |
| ≥5 Years of Follow-Up | 9.30 (1.92 to 45.05) | 0.01 | 1.25 (0.47 to 3.31) | 0.66 |
| **Tumour Size** (T2–T4 vs. in Situ/T0/T1/TX) | 2.91 (1.09 to 7.75) | 0.03 | 2.79 (1.34 to 5.82) | 0.006 |
| **Nodal Involvement** (N1/N2 vs. N0/NX) | 5.89 (1.97 to 17.65) | <0.005 | 1.86 (0.84 to 4.12) | 0.12 |
| **Grading** |  | |  |  |
| Grade 1 | Reference | | | |
| Grade 2 | 0.59 (0.20 to 1.74) | 0.34 | 0.96 (0.31 to 3.02) | 0.95 |
| Grade 3 | 0.23 (0.04 to 1.32) | 0.09 | 1.53 (0.43 to 5.41) | 0.51 |
| **Hormone Receptor Positivity (ER/PR)** | 0.10 (0.03 to 0.43) | <0.005 | 1.08 (0.25 to 4.66) | 0.92 |
| **Age** | 1.01 (0.93 to 1.11) | 0.76 | 1.09 (1.03 to 1.15) | 0.002 |
| **Body Mass Index** (kg/m^2^) | 1.05 (0.94 to 1.17) | 0.41 | 1.07 (0.99 to 1.17) | 0.10 |
| *Cancer-related fatigue was defined based on standardised scores of the EORTC QLQ-C30 fatigue scale (items 10, 12, 18) and the proposed threshold for a clinically important CRF levels as scores ≥39. | | | | |
